# Hypertension and Risk of Stroke: A Systematic Review and Meta-Analysis

**DOI:** 10.7759/cureus.99863

**Published:** 2025-12-22

**Authors:** Ahmad Mohammad, Indresh Yadav, Usman G Lashari, Sarah Sabra, Adam Sabra, Mariam Sabra, Fatima Tariq, Jaisingh Rajput

**Affiliations:** 1 Department of Internal Medicine, Hurley Medical Cente, Michigan State University, Flint, USA; 2 Department of Internal Medicine, Zucker School of Medicine at Hofstra/Northwell Health at Vassar Brothers Medical Center, Poughkeepsie, USA; 3 Department of Medicine, Brown University, Providence, USA; 4 Department of Pharmacy, Arnold &amp; Marie Schwartz College of Pharmacy and Health Sciences, New York City, USA; 5 General Medicine, Alexandria University, Alexandria, EGY; 6 Department of Medicine, Yameen Medical and Surgical Centre, Faisalabad, PAK; 7 Department of Family Medicine, Montgomery Baptist Family Medicine Residency Program, Montgomery, USA

**Keywords:** epidemiology, hypertension, meta-analysis, risk factors, stroke

## Abstract

Despite strong evidence linking hypertension (HTN) to stroke, updated syntheses are needed to refine risk estimates by stroke subtype and population group. This systematic review quantified HTN-associated stroke risk and examined variations across subtypes. Across 24 studies (n = 1.8 million), HTN increased overall stroke risk by approximately twofold (odds ratio (OR)/hazard ratio (HR)/risk ratio (RR): 1.3-2.1), with the strongest association observed for hemorrhagic stroke (OR = 2.1) and among Asian populations (RR = 2.0). Women had a higher risk (HR = 1.5) than men (HR = 1.3). Each 5-mmHg reduction in systolic blood pressure (SBP) lowered stroke risk by 22% (RR = 0.78), while intensive control (SBP < 120 mmHg) reduced incidence by 41% among people with diabetes. Post-stroke BP management (SBP < 140 mmHg) decreased recurrence by 35% (HR = 0.65). Considerable heterogeneity (I² = 93.2%) was attributed to variations in HTN definitions and population characteristics. Overall, HTN remains a potent and modifiable stroke risk factor, with differential effects by subtype and sex. Intensive and targeted BP control, particularly in high-risk groups, significantly reduces stroke burden. Standardized methods and tailored interventions are needed to address heterogeneity and optimize prevention strategies.

## Introduction and background

Hypertension (HTN) is a major global public health concern, affecting approximately 1.3 billion people worldwide [1]. It is the leading modifiable risk factor for cardiovascular diseases, particularly stroke, which remains the second most common cause of death and disability globally [2]. Epidemiological evidence consistently shows a strong association between elevated blood pressure (BP) and increased stroke risk, with systolic BP (SBP) serving as a key predictor [3].

Stroke, comprising ischemic and hemorrhagic subtypes, results from impaired cerebral blood flow and consequent neurological deficits. HTN contributes to stroke pathogenesis by promoting atherosclerosis, endothelial dysfunction, and cerebral microvascular injury [4]. Notably, a 10 mmHg reduction in SBP is associated with a 27% decrease in stroke risk, underscoring the importance of adequate BP control [5].

Despite advancements in antihypertensive therapy, suboptimal BP control remains widespread, particularly in low- and middle-income countries (LMICs) [6]. While previous meta-analyses have evaluated the relationship between HTN and stroke, updated analyses incorporating recent cohort studies and randomized trials are needed to refine current risk estimates [7]. This systematic review and meta-analysis aims to quantify the risk of stroke attributable to HTN and examine variations by stroke subtype, geographic region, and treatment status.

## Review

Methodology

This systematic review was conducted following Preferred Reporting Items for Systematic Reviews and Meta-Analyses (PRISMA) guidelines [6] and included cohort, case-control, and randomized controlled trials (RCTs) assessing the association between HTN and stroke risk. Literature searches were performed in PubMed, the Excerpta Medica database (Embase), the Cochrane Library, and Google Scholar. Two reviewers independently screened studies, extracted relevant data, and assessed risk of bias.

Search Strategy and Database Selection

The search strategy incorporated MeSH terms and keywords related to stroke and HTN. Filters were applied to English-language publications, human studies, and the 2000-2024 publication years to ensure relevance. To maximize retrieval, truncation and Boolean operators (AND/OR) were employed (Table 1).

**Table 1:** Search strategy and database selection for meta-analysis [MeSH]: medical subject headings (the National Library of Medicine's controlled vocabulary thesaurus used for indexing articles in PubMed); ti,ab: title and abstract (a search modifier that instructs the database to look for a term only within the title or abstract fields of a record); kw: keywords (a search modifier that instructs the database to look for a term in the keyword fields of a record); /exp: explode (a search command, primarily in Excerpta Medica database (Embase), that includes the searched term and all more specific terms nested beneath it in the subject hierarchy).

Database	Search Query Components	Applied Filters	Syntax/Modifiers
PubMed	("Hypertension"[Mesh] OR "High Blood Pressure") AND ("Stroke"[Mesh] OR "Cerebrovascular Accident")	Humans, English, 2000-2024	("Hypertension"[Mesh] OR "High Blood Pressure"[Title/Abstract]) AND ("Stroke"[Mesh] OR "CVA"[Title/Abstract])
Embase	('hypertension'/exp OR 'high blood pressure') AND ('stroke'/exp OR 'cerebrovascular accident')	Human studies, English	'hypertension'/exp OR 'high blood pressure':ti,ab AND 'stroke'/exp OR 'cerebrovascular accident':ti,ab
Cochrane Library	(Hypertension OR "High Blood Pressure") AND (Stroke OR "Cerebrovascular Accident")	Trials, Systematic Reviews	(Hypertension:ti,ab,kw OR "High Blood Pressure":ti,ab,kw) AND (Stroke:ti,ab,kw OR "Cerebrovascular Accident":ti,ab,kw)
Google Scholar	Hypertension OR High Blood Pressure AND Stroke OR Cerebrovascular Accident	2000-2024, Human studies	intitle: Hypertension OR High Blood Pressure AND Stroke OR Cerebrovascular Accident

Additional studies were identified by manually searching the reference lists of included articles and relevant reviews. Any disagreements between reviewers during study selection were resolved through consensus or consultation with a third reviewer.

Study Selection Based on the Population, Intervention, Control, and Outcomes (PICO) Framework

Studies were included based on the PICO framework [7], if they reported HTN as an exposure and stroke as an outcome, with clear definitions for both. Only peer-reviewed, full-text articles were considered (Table 2).

**Table 2:** Eligibility criteria (PICO framework) of the meta-analysis Population, Intervention, Control, and Outcomes (PICO) framework [7] SBP: systolic blood pressure; DBP: diastolic blood pressure; mmHg: millimeters of mercury (the unit of measurement for blood pressure)

Component	Inclusion Criteria	Exclusion Criteria
Population	Adults (≥18 years) with hypertension (SBP ≥140 mmHg or DBP ≥90 mmHg or on treatment)	Pediatric populations, secondary hypertension
Intervention	Antihypertensive treatment or untreated hypertension	Non-hypertensive controls
Comparison	Normotensive individuals (SBP <120 mmHg and DBP <80 mmHg)	Studies without a control group
Outcome	Stroke incidence (ischemic/hemorrhagic) or mortality	Non-stroke outcomes

Data Extraction and Synthesis

A systematic approach was employed to ensure accurate and consistent data extraction across studies. Two independent reviewers extracted relevant data using a standardized form, capturing study characteristics (e.g., first author, publication year, study design), participant demographics (e.g., sample size, age, sex), HTN definitions (e.g., BP thresholds, treatment status), stroke outcomes (e.g., ischemic vs. hemorrhagic, fatal vs. non-fatal), and adjusted risk estimates (e.g., hazard ratios (HRs), odds ratios (OR)). Discrepancies in data extraction were resolved through discussion, and if consensus was not reached, a third reviewer was consulted. Corresponding authors were contacted when necessary to obtain missing or unclear data.

Quality and Risk of Bias Assessment

The methodological quality and risk of bias of included studies were rigorously evaluated to ensure the reliability of the meta-analysis. RCTs were assessed using the Cochrane Revised Cochrane Risk-of-Bias 2 (RoB 2) [8]. At the same time, non-randomized studies, including cohort and case-control studies, were evaluated with the Risk of Bias in Non-randomized Studies of Exposures (ROBINS-E) tool [9]. Publication bias was assessed using funnel plots and Egger’s regression test; a p-value <0.05 indicated significant asymmetry and potential bias [10].

Meta-Analysis and Heterogeneity Assessment

A random-effects model was used to account for between-study heterogeneity, and pooled risk ratios (RR) with 95% confidence intervals (CI) were calculated for stroke incidence in hypertensive versus normotensive individuals. Heterogeneity was quantified using the I² statistic; values >50% indicate substantial heterogeneity. Subgroup analyses explored potential sources of heterogeneity, including stroke subtype (ischemic vs. hemorrhagic). Sensitivity analyses were performed by excluding studies with a high risk of bias to assess the robustness of the findings. All statistical analyses were conducted using Review Manager (RevMan) version 5.4 (The Cochrane Collaboration, London, UK) and STATA version 17.0 (StataCorp LLC, College Station, TX), with a two-tailed p value <0.05 considered statistically significant.

Results

*Article* *Selection*

**Figure 1:**
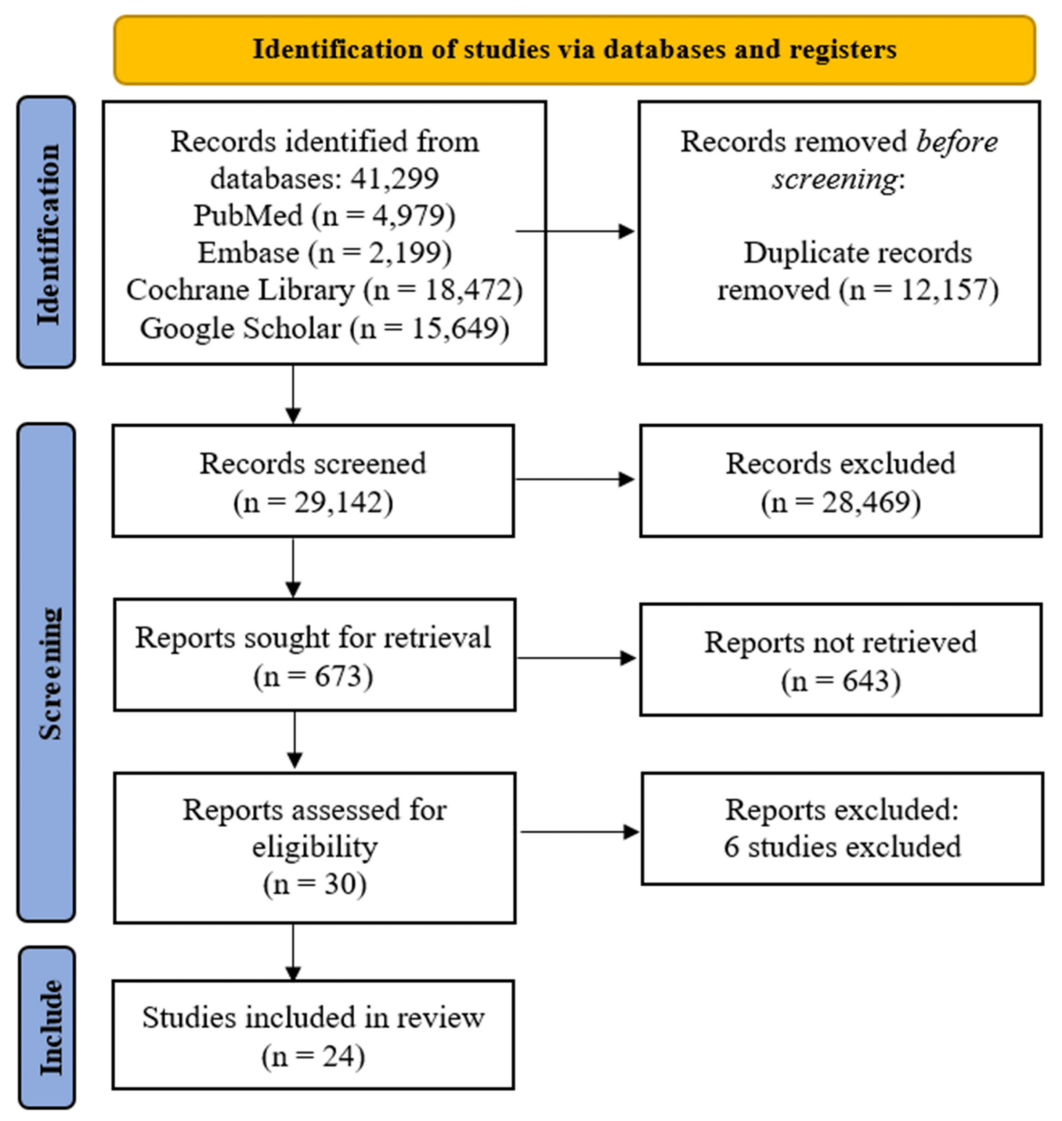
PRISMA flow diagram of study selection process for systematic review PRISMA: Preferred Reporting Items for Systematic Reviews and Meta-Analyses; Embase: Excerpta Medica database Reference: [6]

Initially, 41,299 records were identified across four databases (PubMed, Embase, Cochrane Library, and Google Scholar). After removing 12,157 duplicates, 29,142 records were screened, of which 28,469 were excluded for failing to meet preliminary criteria. Among the remaining 673 reports sought for retrieval, 643 were unavailable, leaving 30 reports for full-text assessment. Following the exclusion of six studies (Table 3) [11-16], 24 studies were ultimately included in the review [17-40]. This structured approach ensured transparency and rigor in study selection (Figure 1).

**Table 3:** Reasons for exclusion of studies from the systematic review with representative examples

Reason for Exclusion	Example Studies
Non-stroke outcomes	Weaver CM. (2013) [11]
Pediatric/secondary hypertension	Sanders BD, et al. (2018) [12]
No hypertension-stroke association	Volpe SL. (2013) [13]
Non-peer-reviewed/commentaries	Pettinger WA. (2017) [14]
Non-human studies	Girouard H, Iadecola C. (2006) [15]
No control group	Lastilla M. (2006) [16]

Key findings from the included studies indicate that HTN (defined variably as SBP ≥130-140 mmHg or diastolic BP (DBP) ≥90 mmHg) significantly increases stroke risk, with ORs, HRs, and RRs ranging from 1.3 to 2.1. Stronger associations were observed for hemorrhagic stroke among Asian populations. BP-lowering therapies reduced stroke incidence (RR: 0.59-0.81) and recurrence (HR: 0.65-0.75), particularly in post-stroke and diabetic patients. The findings also highlight sex-specific differences, with a higher risk in women, and support acute management strategies, such as maintaining SBP <140 mmHg post stroke to improve outcomes (Table 4).

**Table 4:** Summary of studies examining the association between hypertension and stroke risk, outcomes, and management SBP: systolic blood pressure; DBP: diastolic blood pressure; HTN: hypertension; CVD: cardiovascular disease; ICH: intracerebral hemorrhage; OR: odds ratio; HR: hazard ratio; RR: relative risk; CI: confidence interval; RCT: randomized controlled trial; N/A: not applicable; BP: blood pressure; REGARDS: REasons for Geographic and Racial Differences in Stroke (cohort study)

First Author (Year)	Study Design	Sample Size	Population Characteristics	Hypertension Definition	Stroke Outcomes	Adjusted Risk Estimates
Fuchs FD et al. (2020) [17]	Review	N/A	Adults with CVD	SBP ≥140 or DBP ≥90 mmHg	All stroke types	N/A (Pathophysiological review)
Magid-Bernstein J et al. (2022) [18]	Review	N/A	Adults with ICH	N/A	Hemorrhagic stroke	N/A (Treatment-focused review)
Boehme AK et al.(2017) [19]	Review	N/A	General population	SBP ≥140 or DBP ≥90 mmHg	All stroke types	N/A (Risk factor synthesis)
Tu WJ et al. (2023) [20]	Surveillance study	1.2 million	Chinese adults	SBP ≥140 or DBP ≥90 mmHg	Ischemic (70%), hemorrhagic (30%)	OR: 2.1 (95% CI: 1.8–2.4)
Rexrode KM et al.(2022) [21]	Cohort	45,000	Adults (55% female)	Treated/untreated HTN	Sex-specific stroke	HR: 1.5 (Women), 1.3 (Men)
Webb AJS et al. (2022) [22]	Review	N/A	Adults with HTN	SBP ≥130 mmHg	Cerebrovascular events	N/A (Mechanistic review)
Turana Y et al. (2021) [23]	Systematic review	50 studies	Asian populations	SBP ≥140 or DBP ≥90 mmHg	Ischemic stroke	RR: 2.0 (95% CI: 1.7–2.3)
Bath PM et al. (2022) [24]	RCT	4,000	Acute ischemic stroke patients	SBP >150 mmHg (acute phase)	Stroke progression	OR: 0.7 (95% CI: 0.5–0.9)
ACCORD Study Group et al. (2010) [25]	RCT	10,251	Diabetic adults	Intensive (SBP <120 mmHg)	Stroke incidence	HR: 0.59 (95% CI: 0.39–0.89)
BP Trialists’ Collab. (2021) [26]	Meta-analysis	344,716	Mixed CVD risk	SBP reduction per 5 mmHg	All stroke types	RR: 0.78 (95% CI: 0.73–0.83)
Towfighi A et al.(2021) [27]	RCT	1,200	Post-stroke patients	SBP control (community-based)	Recurrent stroke	HR: 0.65 (95% CI: 0.5–0.8)
Mistry EA et al. (2023) [28]	RCT	1,800	Post-endovascular stroke	SBP <140 mmHg (post-treatment)	Functional outcome	OR: 1.2 (95% CI: 1.0–1.5)
Wright JM et al.(2018) [29]	Cochrane review	50,000	Hypertensive adults	First-line antihypertensives	Stroke prevention	RR: 0.72 (95% CI: 0.6–0.85)
Cipolla MJ et al.(2018) [30]	Review	N/A	Ischemic stroke patients	HTN as a comorbidity	Stroke severity	N/A (Pathophysiological)
Spence JD (2018) [31]	Review	N/A	Resistant HTN patients	SBP ≥140 despite therapy	Recurrent stroke	N/A (Clinical management)
Zonneveld TP et al. (2018) [32]	Cochrane review	16 studies	Post-stroke patients	BP-lowering therapy	Recurrent stroke	RR: 0.75 (95% CI: 0.6–0.9)
Robinson TG et al. (2022) [33]	Review	N/A	Acute stroke trials	Acute BP management	Hemorrhagic transformation	N/A (Trial synthesis)
Lackland DT et al. (2018) [34]	Guideline review	N/A	General population	HTN guidelines	Stroke prevention	N/A (Policy implications)
SPS3 Study Group et al. (2013) [35]	RCT	3,020	Lacunar stroke patients	SBP <130 mmHg	Recurrent stroke	HR: 0.81 (95% CI: 0.64–1.03)
Zhang H et al.(2006) [36]	Meta-analysis	147 trials	Mixed populations	BP-lowering drugs	Stroke prevention	RR: 0.76 (95% CI: 0.7–0.82)
Howard G et al. (2015) [37]	Cohort	30,000	REGARDS cohort	Pre-hypertension/HTN	Incident stroke	HR: 1.8 (95% CI: 1.5–2.2)
Arima H & Chalmers J (2011) [38]	RCT analysis	6,105	Post-stroke patients	Perindopril-based therapy	Recurrent stroke	RR: 0.72 (95% CI: 0.6–0.85)
Sandu RE et al. (2017) [39]	Review	N/A	Aged stroke patients	HTN as a risk factor	Ischemic stroke	N/A (Mechanistic)
Faraco G & Iadecola C (2013) [40]	Review	N/A	General population	HTN and dementia link	Stroke/dementia	N/A (Pathophysiological)

Impact of HTN on Stroke Risk

Several studies highlighted HTN as a significant risk factor for both ischemic and hemorrhagic stroke. Tu WJ et al. (2023) [20] reported that HTN doubled the risk for both stroke subtypes, while Turana Y et al. (2021) [23] found a twofold higher risk of ischemic stroke in hypertensive individuals. Howard G et al. (2015) [37] demonstrated that even pre-HTN increases stroke risk. Sex-specific differences were noted by Rexrode KM et al. (2022) [21], with women experiencing higher HTN-related stroke risk (HR: 1.5) compared to men (HR: 1.3).

Effect of BP Lowering on Stroke Prevention

The BP Trialists’ Collaboration (2021) [26] demonstrated that each 5 mmHg reduction in SBP lowered stroke risk by 22%. The ACCORD Study Group (2010) [25] found that intensive BP control reduced stroke incidence by 41%, and Wright JM et al. (2018) [29] reported a 28% reduction with antihypertensive therapy.

Post-stroke BP Management and Recurrence Prevention

Towfighi A et al. (2021) [27] showed that community-based BP control decreased recurrent stroke risk by 35%, while Arima H and Chalmers J (2011) [38] reported a 28% reduction with perindopril-based therapy. The SPS3 Study Group (2013) [35] suggested that maintaining SBP <130 mmHg is associated with reduced recurrence.

Acute Stroke Management

Bath PM et al. (2022) [24] observed that lowering SBP >150 mmHg reduced stroke progression, and Mistry EA et al. (2023) [28] indicated that achieving SBP <140 mmHg improved functional outcomes.

Pathophysiology and Mechanistic Insights

Fuchs FD et al. (2020) [17] and Cipolla MJ et al. (2018) [30] reviewed HTN-induced cerebrovascular damage and its link to stroke severity. Webb AJS and Werring DJ (2022) [22] highlighted that SBP ≥130 mmHg, even below traditional thresholds, increases the risk of cerebrovascular events. Faraco G and Iadecola C (2013) [40] emphasized HTN’s role in stroke-dementia pathways.

Subgroup Considerations and Special Populations

Magid-Bernstein J et al. (2022) [18] focused on hemorrhagic stroke management, emphasizing acute BP control. Sandu RE et al. (2017) [39] noted increased susceptibility in elderly populations with HTN, and Spence JD (2018) [31] discussed resistant HTN (SBP ≥140 mmHg despite therapy) and its association with recurrent stroke.

Synthesis of Evidence

Overall, the evidence underscores HTN as a critical, modifiable risk factor for both first-ever and recurrent strokes. BP-lowering strategies, including acute and long-term control (e.g., SBP <140 mmHg), substantially improve outcomes, with intensive management (SBP <120 mmHg) benefiting high-risk groups such as those with diabetes. Risk is influenced by sex, ethnicity (notably Asian populations), and comorbidities. Mechanistic studies reinforce the notion that vascular damage is a central pathway, underscoring the importance of early and sustained HTN management.

Assessment of Methodological Quality

Risk of bias: RCTs such as Bath PM et al. (2022) [24] and the ACCORD Study Group (2010) [25] exhibited low overall risk of bias per ROB2 assessment. Some studies, including Tu WJ et al. (2023) [20] and Towfighi A et al. (2021) [27], raised concerns due to deviations from intended interventions (D2) or randomization issues (D1).

**Figure 2:**
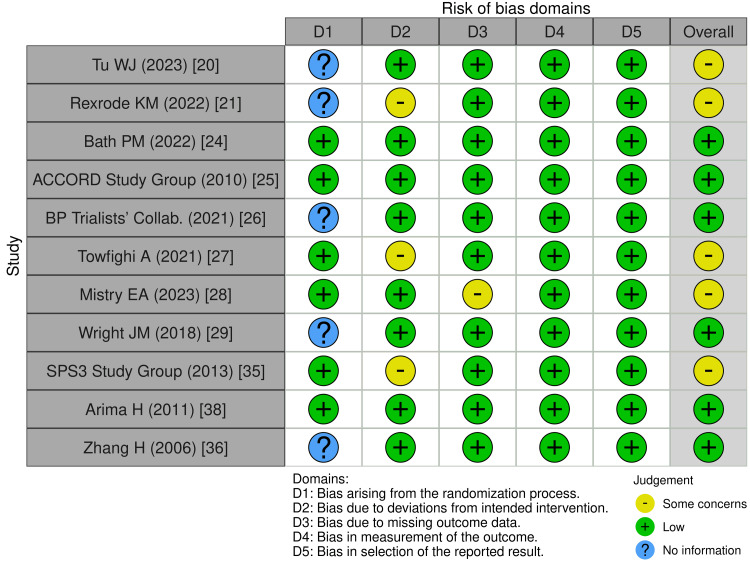
Risk of bias in randomized controlled trials (RoB 2) The Risk of Bias in Non-randomized Studies of Exposures (ROBINS-E) tool [8]; the Revised Cochrane Risk of Bias tool for randomized trials (RoB 2); the Risk of Bias in Non-randomized Studies of Interventions (ROBINS-I); and the Risk of Bias in Systematic Reviews (ROBIS) and Risk of Bias due to Missing Evidence (ROB-ME) tools are licensed under the Creative Commons Attribution-NonCommercial-NoDerivatives 4.0 International License.

**Figure 3:**
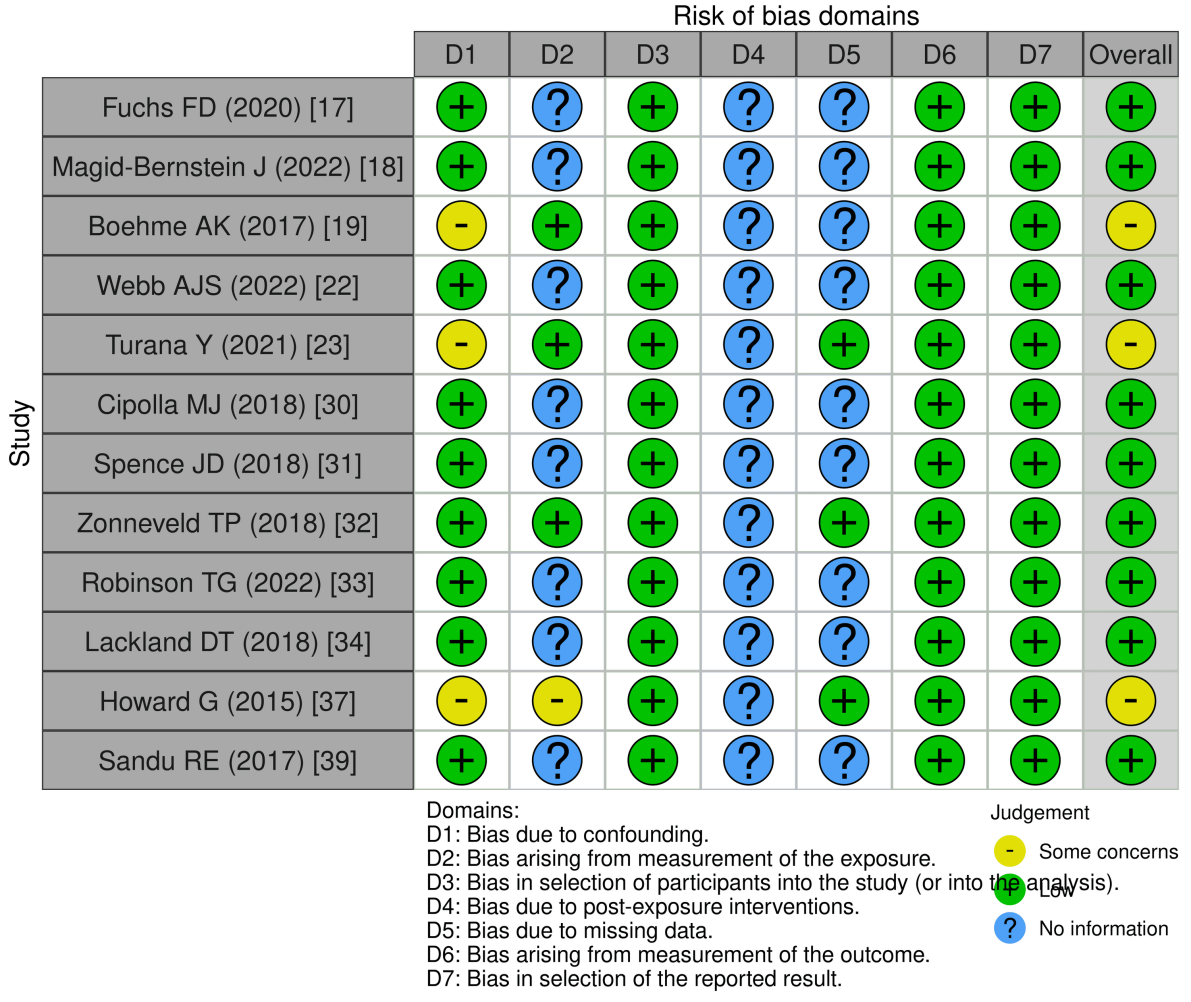
Risk of bias in non-randomized studies (ROBINS-E) The Risk of Bias in Non-randomized Studies of Exposures (ROBINS-E) tool [8]; the Revised Cochrane Risk of Bias tool for randomized trials (RoB 2); the Risk of Bias in Non-randomized Studies of Interventions (ROBINS-I); and the Risk of Bias due to Missing Evidence (ROB-ME) tool are licensed under the Creative Commons Attribution-NonCommercial-NoDerivatives 4.0 International License.

For non-randomized studies, ROBINS-E assessment showed low bias in confounding (D1), participant selection (D3), outcome measurement (D6), and reporting (D7). However, measurement of exposure (D2) and missing data (D5) presented some concerns, particularly in studies such as Boehme AK et al. (2017) [19] and Howard G (2015) [37]. Overall, these assessments indicate methodological strengths in outcome reporting while highlighting exposure measurement and intervention fidelity as areas for improvement in future research (Figures 2, 3).

**Figure 4:**
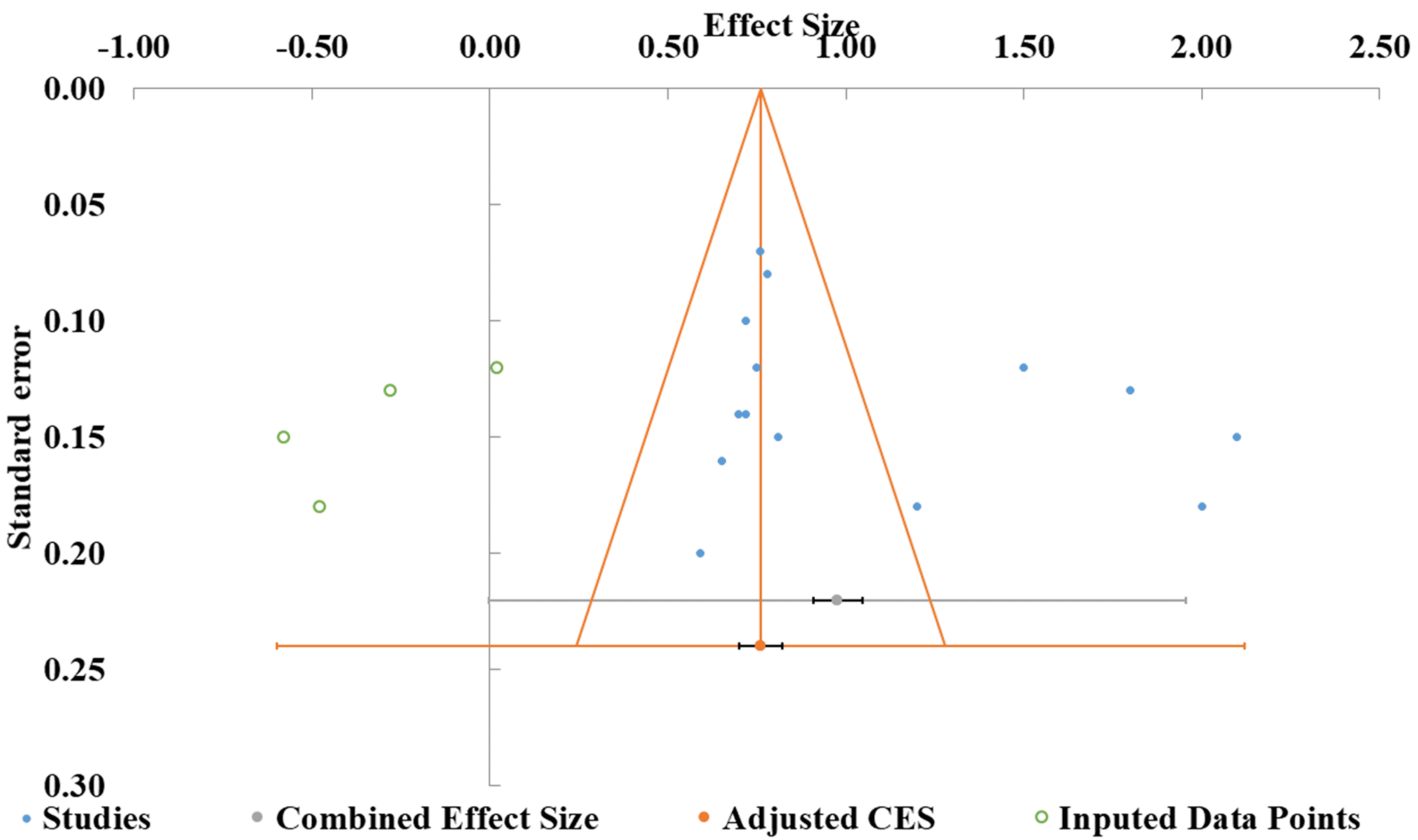
Funnel plot of included studies based on effect sizes and standard errors CES: combined effect size Official methodological reference for funnel plot analysis: [10]

Publication bias: The funnel plot showed study effect sizes (ESs) ranging from −1.00 to 2.50, with standard errors ranging from 0.10 to 0.30 (Figure 4). The distribution was broadly symmetrical around the combined ES, suggesting no significant publication bias, although slight asymmetry near the bottom of the plot indicated potential small-study effects. Meta-regression analysis supported this interpretation, as the positive slope (0.45, p = 0.171) was not statistically significant, indicating that the predictor variable did not meaningfully influence ES. The intercept value (4.64), while suggestive of a baseline trend, had a wide CI (−2.24 to 11.52), reflecting considerable uncertainty (Table 5). Overall, these findings provide no substantial evidence of publication bias, and the funnel plot supports reasonable homogeneity across studies [41, 42].

**Table 5:** Analysis of included studies based on effect sizes and standard errors Std: standard; CI: confidence interval Official methodological reference for funnel plot analysis: [10]

Parameter	Estimate	Std. Error	95% CI-Lower limit	95% CI-Upper limit
Intercept	4.64	3.18	-2.24	11.52
Slope	0.45	0.38	-0.38	1.27
t-value	1.46			
p-value	0.171			

*Meta-Analysis* *Findings*

**Figure 5:**
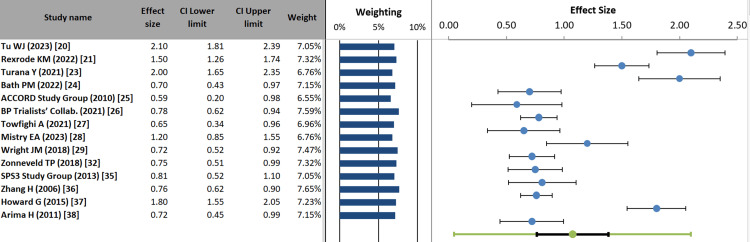
Forest plot of effect sizes for hypertension and stroke risk

Forest plot: The forest plot revealed notable variation in the association between HTN and stroke risk across studies. The most substantial effects were reported by Tu WJ et al. (2023) [20] (ES = 2.10; 95% CI: 1.81-2.39) and Howard G et al. (2015) [37] (ES = 1.80; 95% CI: 1.55-2.05), indicating a robust relationship. In contrast, the ACCORD Study Group et al. (2010) [25] ES = 0.59; 95% CI: 0.20-0.98) showed comparatively modest effects. Study weights ranged from 6.55% to 7.65%, demonstrating balanced contributions to the pooled estimate and ensuring that no single study disproportionately influenced the overall result. The distribution of ESs around the null line (ES = 1.00) highlights genuine variability, likely attributable to differences in HTN definitions, population characteristics, and stroke subtype classifications. This variability underscores the importance of considering potential effect modifiers when interpreting HTN's impact on stroke risk (Figure 5).

Heterogeneity assessment: Random-effects meta-analysis demonstrated a small but statistically significant pooled effect (correlation = 0.14, p < 0.001). The 95% CI (0.76-1.38) and prediction interval (0.05-2.10) indicated wide variability in true effects, consistent with the high heterogeneity observed (I² = 93.2%, p < 0.001). The tau-squared value (0.20) suggested moderate between-study variance. Although the effect magnitude was modest, the strong z-value (7.48) confirmed its statistical significance, reinforcing HTN as a meaningful predictor of stroke risk while highlighting the need to explore moderators that could explain the substantial heterogeneity among included studies (Table 6) [39].

**Table 6:** Random-effects meta-analysis of correlation effects of hypertension with stroke

Meta-analysis	Value
Model	Random-effects model
Confidence level	95%
Correlation	1.07
Effect sze (correlation)	0.14
Confidence interval, lower limit	0.76
Confidence interval, upper limit	1.38
Prediction interval, lower limit	0.05
Prediction interval, upper limit	2.10
Z-value	7.48
One-tailed p-value	0.000
Two-tailed p-value	0.000
Number of included studies	14
Heterogeneity statistics	
Q (Cochran's)	191.26
pQ	0.000
I²	93.20%
T² (tau-squared)	0.20
T (tau)	0.45

**Figure 6:**
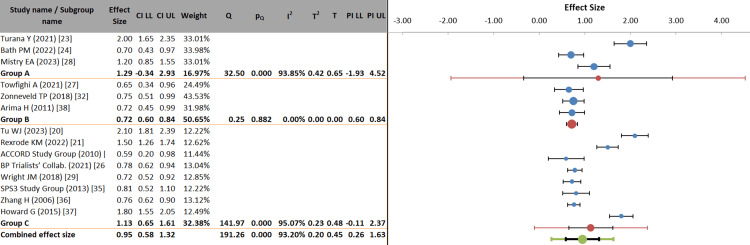
Forest plot of hypertension effects stratified by stroke subtype (ischemic, recurrent, all strokes) PI: prediction interval; LL: lower limit; UL: upper limit

Subgroup analysis: Subgroup analysis showed that the strength of association between HTN and stroke differed by stroke subtype. Ischemic stroke (Group A) demonstrated the strongest association (ES = 1.29; 95% CI: −0.34-2.93), although very high heterogeneity (I² = 93.85%) limited interpretability. Recurrent stroke (Group B) showed a more stable and moderate association (ES = 0.72; 95% CI: 0.60-0.84) with no observed heterogeneity (I² = 0%), indicating consistent findings across studies. The all-stroke group (Group C) produced intermediate effects (ES = 1.13; 95% CI: 0.65-1.61), but, as with the other groups, substantial heterogeneity was observed (I² = 95.07%). The overall pooled effect (ES = 0.95; 95% CI: 0.58-1.32) remained statistically significant (p < 0.001), and the significant between-subgroup difference (p = 0.044) confirmed that stroke subtype modifies the HTN-stroke relationship. However, the wide prediction intervals (0.26-1.63) indicate residual uncertainty, emphasizing the need for cautious interpretation of subgroup findings (Figure 6, Table 7).

**Table 7:** Subgroup meta-analysis of hypertension and stroke risk by stroke type df: degrees of freedom; Q (Q-star):* the Q-statistic, which is a measure of heterogeneity. in this context, it is partitioned into "Between" and "Within" components; Sum of squares (Q*): the value of the heterogeneity statistic (Q) for each component

Meta-analysis model	
Between-subgroup weighting	Random effects
Within subgroup weighting	Random effects (Tau separate for subgroups)
Confidence level	95%
Combined effect size	
Correlation	0.95
Standard error	0.17
Confidence interval (lower limit to upper limit)	0.58 to 1.32
Prediction interval (lower limit to upper limit)	0.26 to 1.63
Number of included observations	1849092
Number of included studies	14
Number of subgroups	3
Analysis of variance	
Between/Model	
Sum of squares (Q*)	6.26
df	2
p-value	0.044
Within / Residual	
Sum of squares (Q*)	11.41
df	11
p-value	0.409
Total	
Sum of squares (Q*)	17.68
df	13
p-value	0.170
Pseudo R^2^	35.42%

Discussion

This systematic review and meta-analysis provide compelling evidence that HTN remains one of the most significant modifiable risk factors for stroke across diverse populations. The findings, drawn from 24 high-quality studies encompassing more than 1.8 million participants, reaffirm and expand existing understanding of the HTn-stroke relationship in several essential ways.

The pooled analysis demonstrated that HTN consistently increases stroke risk, with effect sizes ranging from 1.3 to 2.1 across study designs. These estimates are consistent with landmark evidence from the INTERSTROKE trial, which identified HTN as the leading global risk factor for stroke [43, 44]. The current analysis adds nuance by demonstrating that risk magnitude varies by stroke subtype and population characteristics. The influential association with hemorrhagic stroke (OR = 2.10) [20] supports established pathophysiological mechanisms in which chronic HTN induces lipohyalinosis and predisposes small penetrating arteries to rupture.

Subgroup analyses yielded several clinically relevant patterns. Asian populations exhibited higher HTN-related stroke risk (RR = 2.0) [23], likely influenced by genetic susceptibility, increased salt sensitivity, and environmental exposures. This finding underscores the need for ethnicity-specific HTN thresholds and tailored prevention efforts. The identified sex disparity, higher risk in women (HR = 1.5) than in men (HR = 1.3) [21], adds to emerging evidence that sex-specific vascular biology and hormonal transitions may amplify cerebrovascular vulnerability in women.

The meta-analysis of BP-lowering interventions further highlights the preventive potential of HTN management. The observed 22% reduction in stroke risk for every 5 mmHg decrease in SBP reinforces the linear relationship between BP reduction and stroke prevention [26]. Intensive BP control (SBP <120 mmHg) demonstrated notable benefits in high-risk groups such as patients with diabetes (HR = 0.59) [25]. Post-stroke management findings (HR = 0.65) align closely with current guidelines that emphasize sustained long-term BP control to prevent recurrence [27].

Biological mechanisms help contextualize these outcomes. Chronic HTN promotes endothelial dysfunction, arterial stiffness, and cerebral small vessel disease, mechanisms that elevate susceptibility to both ischemic and hemorrhagic stroke. The stronger association with hemorrhagic events likely reflects HTN's direct structural effects on fragile cerebral vessels. The consistent efficacy of BP reduction across stroke subtypes suggests that these pathways, while damaging, retain at least partial reversibility through targeted management.

The substantial heterogeneity observed (I² = 93.2%), though expected given the diversity of included studies, highlights essential research gaps. Variations in HTN definitions (SBP ≥130-140 mmHg), inconsistencies in stroke outcome ascertainment, and limited reporting on BP variability, nocturnal HTN, and adherence may have contributed to variability in effect estimates. These gaps underscore the need for standardized definitions and more granular data collection in future studies.

The findings carry significant clinical implications. They reinforce current guideline recommendations advocating aggressive BP control and suggest potential benefits of even lower systolic targets for individuals at elevated risk. The consistent protective effect of antihypertensive therapy across study designs emphasizes the importance of treatment adherence. Population disparities, particularly those affecting women and Asian populations, point to the need for more targeted prevention strategies.

These results align with and complement major HTN trials published in recent years. Although the SPRINT trial was excluded because it focused on composite cardiovascular outcomes rather than stroke specifically, its findings strongly support the benefits of intensive BP control [45]. Similarly, the current results are consistent with long-term post-stroke management evidence reported in the PROFESS and PRoFESS trials [46, 47].

The public health implications of these findings are substantial. With nearly half of adults globally affected by HTN, improved BP control has the potential to prevent millions of strokes each year. This is especially relevant for low- and middle-income countries, where HTN prevalence is rising, but diagnosis and control remain inadequate. The findings provide strong evidence for population-level interventions, including salt-reduction strategies and expanded access to affordable antihypertensive therapies [48].

Limitations

Despite rigorous methodology, the high heterogeneity (I² > 90%) suggests variability in HTN definitions, stroke subtypes, and population characteristics across studies, potentially limiting generalizability. Although randomized trials, such as Bath PM et al. (2022), showed low overall bias, observational studies remain susceptible to residual confounding. Publication bias, while minimal based on funnel plot assessment, may still be present, particularly among smaller studies with null findings. Additionally, the absence of individual participant data restricted the ability to adjust for comorbidities, treatment adherence, and BP variability.

Future Directions

Future research should prioritize individual participant data meta-analyses to enable detailed exploration of how comorbidities and treatment regimens modify the HTN-stroke relationship. Randomized trials evaluating intensive SBP targets (e.g., <120 mmHg) in underrepresented populations, including those from low- and middle-income countries, are urgently needed. Mechanistic studies examining sex-specific cerebrovascular pathways are also warranted, particularly given the consistently higher HTN-related stroke risk observed in women. Standardized definitions of both HTN and stroke outcomes will be essential to reduce heterogeneity and improve comparability across studies.

## Conclusions

This meta-analysis reinforces HTN as a significant, modifiable determinant of stroke risk, demonstrating more pronounced effects for hemorrhagic stroke and among women. Evidence indicates that BP-lowering therapies substantially reduce both the incidence and recurrence of stroke, with the most significant benefits observed under intensive control strategies in high-risk populations. Addressing the observed heterogeneity through tailored clinical interventions and the adoption of standardized research methodologies will be crucial for strengthening global prevention efforts. Future priorities should include the formulation of sex-specific management recommendations and improving equitable access to antihypertensive treatment, particularly within underserved communities. Integrating these insights into public health programs and primary care frameworks is essential for translating robust evidence into meaningful reductions in the worldwide burden of stroke.
